# Deep Learning–Based Identification of Tissue of Origin for Carcinomas of Unknown Primary Using MicroRNA Expression: Algorithm Development and Validation

**DOI:** 10.2196/56538

**Published:** 2024-07-24

**Authors:** Ananya Raghu, Anisha Raghu, Jillian F Wise

**Affiliations:** 1 Quarry Lane School San Ramon, CA United States; 2 Department of Biology and Biomedical Sciences Salve Regina University Newport, RI United States; 3 Broad Institute of MIT and Harvard Cambridge, MA United States; 4 Pre-College Programs Tufts University Medford, MA United States

**Keywords:** cancer genomics, machine learning algorithms, deep learning, gene expression, RNA, RNAs, cancer, oncology, tumor, tumors, tissue, tissues, metastatic, microRNA, microRNAs, gene, genes, genomic, genomics, machine learning, algorithm, algorithms, carcinoma, genetics, genome, detection, bioinformatics

## Abstract

**Background:**

Carcinoma of unknown primary (CUP) is a subset of metastatic cancers in which the primary tissue source of the cancer cells remains unidentified. CUP is the eighth most common malignancy worldwide, accounting for up to 5% of all malignancies. Representing an exceptionally aggressive metastatic cancer, the median survival is approximately 3 to 6 months. The tissue in which cancer arises plays a key role in our understanding of sensitivities to various forms of cell death. Thus, the lack of knowledge on the tissue of origin (TOO) makes it difficult to devise tailored and effective treatments for patients with CUP. Developing quick and clinically implementable methods to identify the TOO of the primary site is crucial in treating patients with CUP. Noncoding RNAs may hold potential for origin identification and provide a robust route to clinical implementation due to their resistance against chemical degradation.

**Objective:**

This study aims to investigate the potential of microRNAs, a subset of noncoding RNAs, as highly accurate biomarkers for detecting the TOO through data-driven, machine learning approaches for metastatic cancers.

**Methods:**

We used microRNA expression data from The Cancer Genome Atlas data set and assessed various machine learning approaches, from simple classifiers to deep learning approaches. As a test of our classifiers, we evaluated the accuracy on a separate set of 194 primary tumor samples from the Sequence Read Archive. We used permutation feature importance to determine the potential microRNA biomarkers and assessed them with principal component analysis and t-distributed stochastic neighbor embedding visualizations.

**Results:**

Our results show that it is possible to design robust classifiers to detect the TOO for metastatic samples on The Cancer Genome Atlas data set, with an accuracy of up to 97% (351/362), which may be used in situations of CUP. Our findings show that deep learning techniques enhance prediction accuracy. We progressed from an initial accuracy prediction of 62.5% (226/362) with decision trees to 93.2% (337/362) with logistic regression, finally achieving 97% (351/362) accuracy using deep learning on metastatic samples. On the Sequence Read Archive validation set, a lower accuracy of 41.2% (77/188) was achieved by the decision tree, while deep learning achieved a higher accuracy of 80.4% (151/188). Notably, our feature importance analysis showed the top 3 most important features for predicting TOO to be microRNA-10b, microRNA-205, and microRNA-196b, which aligns with previous work.

**Conclusions:**

Our findings highlight the potential of using machine learning techniques to devise accurate tests for detecting TOO for CUP. Since microRNAs are carried throughout the body via extracellular vesicles secreted from cells, they may serve as key biomarkers for liquid biopsy due to their presence in blood plasma. Our work serves as a foundation toward developing blood-based cancer detection tests based on the presence of microRNA.

## Introduction

Carcinoma of unknown primary (CUP) originates when a patient presents at diagnosis with malignant disease across the body; yet, the cancer cells tissue of origin (TOO) remains unidentifiable. Thus, CUP is a unique subset of metastasized cancer representing an advanced stage in which cancer has gained the ability to thrive in new tissue sites and has spread from the primary tumor site. In the United States, an estimated 31,490 people were diagnosed with cases of cancer of unknown TOO in 2008. This accounts for nearly 3%-5% of all cancer cases [1] and given the lack of knowledge on tissue response to current therapeutics the median survival of patients remains only 3-9 months [2]. In many cases of CUP, the primary site is never identified, preventing the use of treatment that can be effective for the true TOO [3,4]. It has been demonstrated that pinpointing the primary site can significantly increase survival rates by enabling precise and targeted treatment [5].

Unfortunately, primary tumor identification poses various challenges. Techniques such as serum tumor markers and imaging tests are used to identify the TOO, although only 30% of these tests are successful. Moreover, some positive findings can be misleading [6] and CUP diagnostic workups are often time-consuming, expensive, and unsuccessful [7]. These difficulties have spurred interest in using genetic expression data, such as microRNA, to identify the TOO.

MicroRNAs belong to a class of noncoding regulatory RNAs, small single-stranded RNA molecules that are between 19 and 25 nucleotides long and are involved in the regulation of gene expression of mRNAs. MicroRNAs hold promise as informative biomarkers for cancer due to their significant involvement in cellular processes such as cell division, apoptosis, proliferation, and oncogenesis [8]. Beyond their intracellular role in gene regulation, microRNAs may be carried throughout the body via extracellular vesicles secreted from cells and have been identified in the blood. Additionally, microRNA, unlike mRNA, is characterized by resistance to extreme temperatures and pH. This makes microRNAs far more stable biomarkers [9,10].

Previous work [11] demonstrates that microRNA expression is more informative in classifying tumor samples by their origin in comparison to mRNA. Specifically, microRNAs are better at classifying poorly differentiated tumors [12]. Moreover, microRNAs have shown great potential for identifying TOO for cancers of unknown primary origin [13]. MicroRNAs have been investigated as prognostic and diagnostic biomarkers extensively in the research community and have even been found to be deregulated in numerous cancers [14].

With the wide availability of large data sets containing gene expression data, computational techniques such as machine learning have emerged as promising tools for improving TOO detection. Machine learning implementations have increased accuracy in predicting cancer and have the potential to improve the diagnosis, prognosis, and therapy selection for patients with cancer [15]. The 3 traditional machine learning models are decision trees, random forests, and logistic regression. Decision trees [16] attempt to partition the training set into subsets that contain samples of only one class, thereby predicting the class of interest. Random forests are ensemble classifiers, combining multiple trees for higher accuracy [17]. In contrast, logistic regression is a predictive algorithm to find a model that can predict categorical output [18]. Deep learning is a subset of machine learning designed to mimic the human brain through the use of artificial neural networks by using many layers and larger data sets. Generally, deep learning techniques are well suited for discovering and recognizing complex patterns in data that traditional machine learning methods can often miss. The increasing incorporation of deep learning in health care along with the availability of highly characterized cancer data sets has further accelerated research into the applications of deep learning in the analysis of the biology of cancer [19].

Given the complexities of diagnosing a TOO from a cancer that has spread throughout the body, previous investigators have applied machine learning methods to determine TOO for metastasized cancers [20,21]. Longstanding techniques of microarrays and polymerase chain reaction have been used for the generation of machine learning models for CUP detection, including support vector machines with 89% accuracy [22] and the k-nearest neighbor algorithm with 82% accuracy [23,24]. LoCUP, a TOO classifier, was the first machine learning model using a multinomial logistic regression classifier with ridge penalties to incorporate tumor purity and reached a 95.8% accuracy [25]. Cup AI Dx [20] used mRNA gene expression data from The Cancer Genome Atlas (TCGA) data set to train a network based on the popular inception model [22] to identify the TOO, achieving an accuracy of 96.7% on a validation set of 354 TCGA metastatic samples. The TOD-CUP method [21] addressed the variation in mRNA platforms and used a gene expression rank–based majority vote algorithm to achieve an overall accuracy of 94%. Early work with microRNAs and nondeep learning machine learning algorithms showed 84% accuracy with k-nearest neighbor models [26] and binary decision trees at 85% [27]. However, the investigation of deep learning machine learning models may improve these accuracies with TOO detection by microRNA. MicroRNAs are also at the forefront of extracellular vesicle liquid biopsy development and may be better suited for the noninvasive classification of TOO [28].

This study sets out to explore the possibility of developing a model for using microRNA profiles from metastatic tissues to determine the TOO through the application of deep learning techniques. Successful TOO detection from microRNAs will provide a route for cancer detection without requiring samples from the primary tumor site in cases of CUP malignancies. We hypothesize that we would be able to predict the origin of metastatic tumors with higher accuracy than previous reports by leveraging larger data sets of microRNA profiles from both normal and primary site tissues to train the model.

The data for this project were collected from TCGA data set [29] and the Sequence Read Archive (SRA) [30] from microRNA tissue expression database. The TCGA data set contains samples from 18 different cancer types representing 9648 samples, of which 365 were metastatic, 633 were solid normal, and 8650 were from the primary tumor site. Each sample consisted of microRNA expression data, available as RPM (reads per million mapped reads), as well as metadata including age and gender. We split TCGA data set into a combined primary tumor or solid normal samples training set and a metastatic sample test set. We then further split the primary tumor and solid normal samples into a training and validation set with a 9:1 ratio. The training set consisted of 8355 samples and the validation set consisted of 928 samples.

We use 2 data sets for evaluating the performance of our models. The SRA test data set consisted of 194 samples from 5 different cancer types, all of which were from the primary tumor. We also used the metastatic samples from TCGA data set as our final test data set, which contained samples from 6 cancer types. We developed 4 machine learning models, a decision tree classifier, random forest, logistic regression, and finally, a deep learning model. Our deep learning model performed with the highest accuracy, achieving an accuracy of 97% in detecting TOO for metastatic samples and 80.4% on the nonmetastatic SRA cohort. Feature importance analysis revealed the top 3 differentiating microRNA targets as microRNA-10b, microRNA-196b, and microRNA-205, which confirms prior investigations on microRNAs associated with metastatic cancer [31-33].

## Methods

### Data Sets

**Figure 1:**
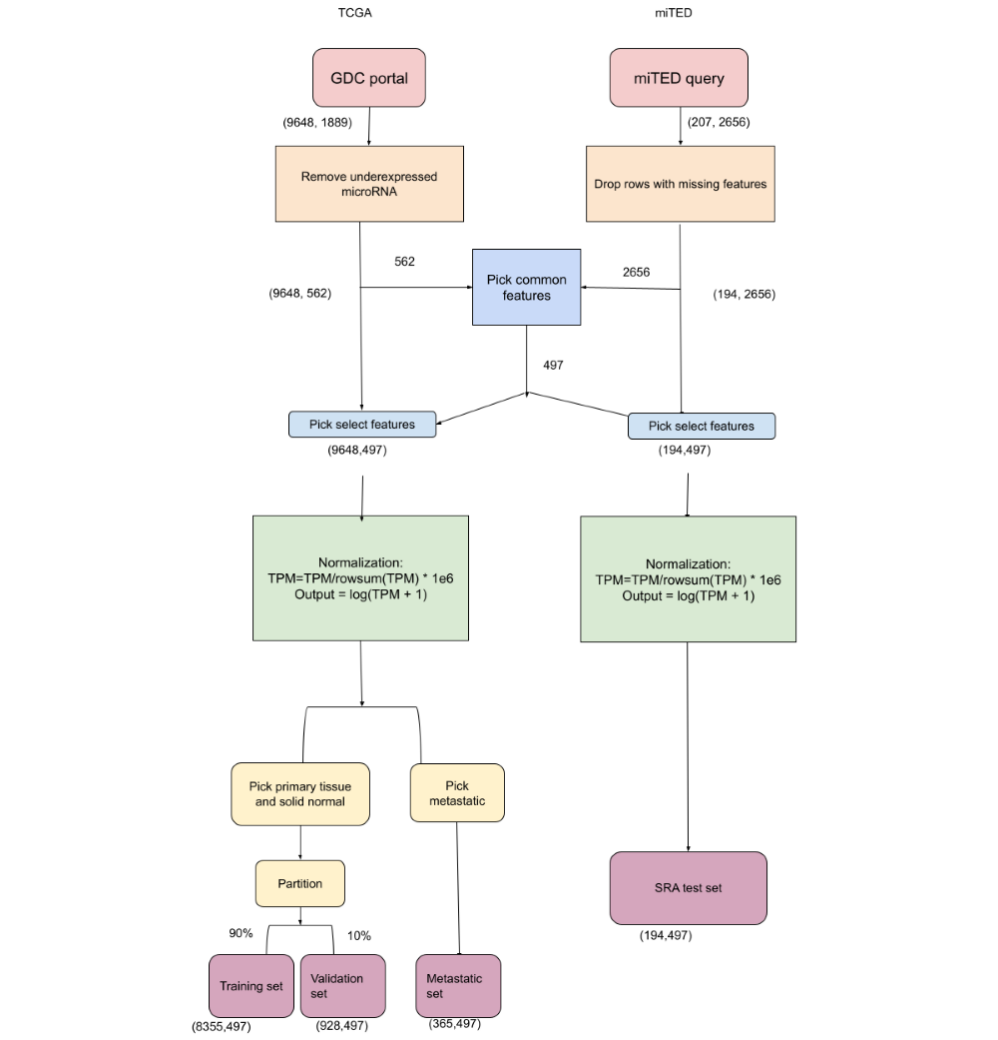
Overview of our data processing pipeline. Data from the TCGA GDC portal and SRA miTED portal was obtained. Underexpressed microRNA and samples containing missing features from the miTED data were filtered. Common features were selected between both data sets, reducing the number of microRNA to 497. Features were normalized as reads per million per sample and log-transformed. TCGA data set was split into (1) the primary tissue and solid normal set and (2) the metastatic test set. The first, combined, set was further split into a training and validation set. GDC: Genomics Data Commons; miTED: microRNA tissue expression database; SRA: Sequence Read Archive; TCGA: The Cancer Genome Atlas.

**Figure 2:**
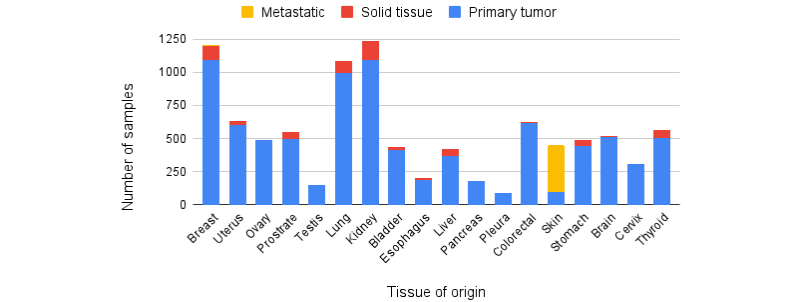
The Cancer Genome Atlas (TCGA) data set distribution across tissue of origin (TOO). Distribution of the different cancer samples in the TCGA data set that are from the primary tumor site, solid tissue, or metastatic. Note that metastatic samples primarily corresponded to the skin as the TOO.

In Figure 1, we outline the data preprocessing pipeline. Our study analyzed published data and did not generate any new sequencing data. TCGA data were obtained [29]. Data were further filtered by querying the Genomics Data Commons via the Application Programming Interfaces specified [34]. We restricted the tissue type to be one of the primary tumors, solid tissue normal, or metastatic. We further restricted the data to microRNA transcriptome profiling and picked data corresponding to 18 types of cancer each containing a sufficient number of samples, obtaining 9648 files (Figure 2 and Table S1 in Multimedia Appendix 1).

To obtain the SRA data, we used the microRNA tissue expression database portal and restricted the cancer types to 6 types of cancer, seen in further detail in Figure 2. We obtained 207 samples, each containing expression data for 2656 microRNAs. After removing samples with missing features, 194 samples were remaining.

We selected microRNA features that were expressed in at least 50% (4824/9648) of the samples, which reduced the number of features in the TCGA data set from 1889 to 562. We then picked the common features between the SRA data set and the TCGA data set, reducing this number to 497. On both data sets, we normalized the RPM of the selected features per sample to sum to a million. We then transformed the RPM values using the transformation log(RPM + 1) to restrict the range of the input.

### Training Procedure

For the implementation of decision tree, random forest, and logistic regression classifiers, the sklearn package was used [35]. We used classification accuracy as the primary metric to evaluate our models. Deep learning models were created with PyTorch (Meta AI) [36]. To optimize and train our neural network, we used Adam optimizer and trained for 50 epochs. Since our objective was classification, we used softmax with cross-entropy loss [37] to optimize the model. We used the validation set to determine the hyperparameters of the models and picked the best-performing model for further evaluation on the test set. Feature importance was calculated with sklearn’s permutation feature importance function.

### Ethical Considerations

This study was conducted in accordance with the ethical standards of the Salve Regina University ethical standards. The research study was reviewed by the institutional review board of Salve Regina University and was determined to be exempt from further review as per criteria contained in Title 45 CFR §46.104(d) section 4ii of federal regulations. As such, the study used only publicly available deidentified or anonymized data, and the project was reviewed (Exemption #Wise.2024.6.11).

## Results

In order to develop a model to detect TOO, we set out to find the best-performing machine learning model for determining the TOO from the TCGA primary tumor and solid normal tissue cohorts. The models were then tested on the validation set, and we could accurately determine the TOO based on primary or normal microRNA profiles, with an accuracy of over 90% for 15 of 18 different tissue types using deep learning (Table 1 and Table S2 in Multimedia Appendix 1).

We can note that the deep learning model performs consistently the highest on the validation set, with logistic regression and random forest classifiers providing comparable performance.

We then set out to apply our best-performing deep learning model and evaluate its performance on the SRA test set that contains microRNA expression data from primary tumors (Table 2). We accurately determined the TOO with an accuracy of over 90% (90/100) for 3 of the 5 cancer types but saw a decrease in accuracy for bladder and colorectal cancer.

Finally, we analyzed our deep learning model on microRNA expression data from metastatic tissue samples in the TCGA data set (Table 3). We accurately determined the TOO with an accuracy of over 85% (308/362) for all cancer types with an average of 97% (351/362).

Since random forest and logistic regression classifiers provided comparable performance on the primary or normal validation set, we compared the classifier accuracy on both test sets for all created models (Table 4).

**Figure 3:**
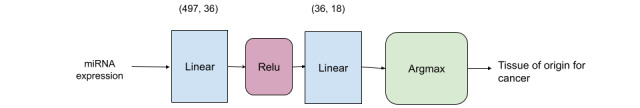
A schematic of the machine learning model architecture. MiRNA: microRNA.

The input features of our models consist of microRNA expression data common to TCGA and SRA data sets. Figure 3 describes the overall architecture of the model, which consists of 2 linear layers. The second layer has 18 outputs, corresponding to each cancer type. The cancer type corresponds to the output with the maximum value.

We used dropout for the input layer [38] as it is a common technique to improve model accuracy and reduce overfitting. We also augmented our input data with noise.

**Figure 4:**
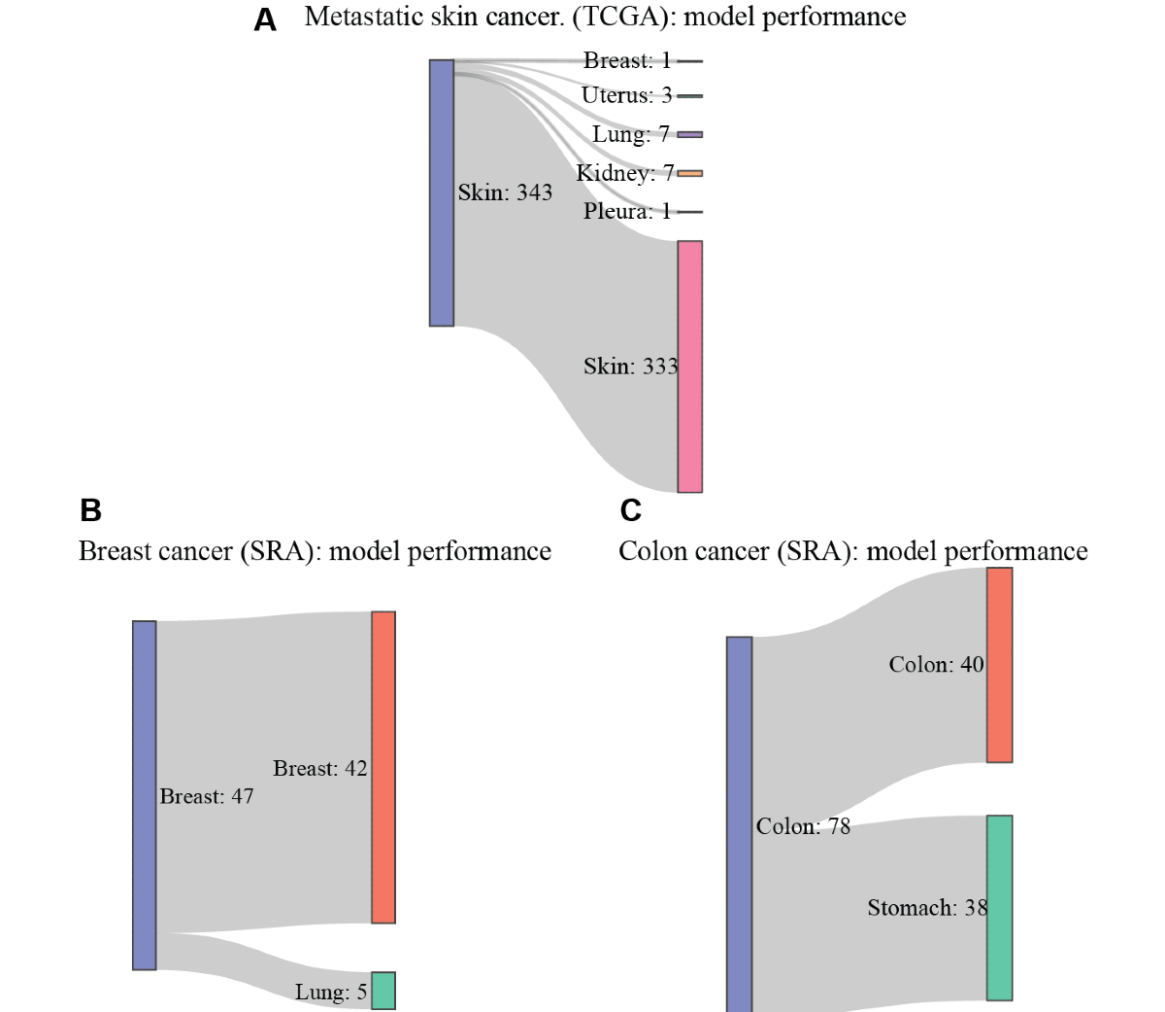
Sankey plot for deep learning model on Sequence Read Archive (SRA) and The Cancer Genome Atlas (TCGA) test data sets. (A) On the TCGA data set, our deep learning model is able to correctly classify 333 out of 343 metastatic skin cancer samples, demonstrating high accuracy. (B) On the SRA test data set, we show representative plots for breast and colon cancers, showing high accuracy for breast cancer tissue of origin identification. (C) The model performance on colon cancer is less accurate due to microRNA expression consistently overlapping for colon and stomach cancers [40].

To evaluate the performance of our models, we computed confusion matrices for performance on metastatic samples (Figure S2A and S2B in Multimedia Appendix 1) and plotted the receiver operating characteristic curves for performance on metastatic skin cancer (Figure S2C and S2D in Multimedia Appendix 1), as the majority of the metastatic samples were obtained from skin cancer cases. We observed that the deep learning model performed significantly better than our decision tree model, which was consistent when evaluated on the SRA validation cohort (Figure S3 in Multimedia Appendix 1). To illustrate the effectiveness of our models, we created Sankey plots representing the deep learning model performance on metastatic samples from the TCGA data set and primary tissue sites from the SRA data set (Figure 4).

**Table 1:** Model accuracies on the validation test set. Performance of 4 models for the identification of tissue of origin. The validation set consists of both primary tumor and solid normal tissue samples from The Cancer Genome Atlas data set.

Cancer type	Decision tree (%)	Random forest (%)	Logistic regression (%)	Deep learning (%)
Breast (n=131)	91.6	99.2	96.9	99.2
Uterus (n=73)	76.7	100	90.4	94.5
Ovary (n=48)	89.6	91.6	93.8	100
Prostrate (n=54)	94.5	100	100	100
Testis (n=18)	61.1	94.5	94.4	88.9
Lung (n=117)	81.1	95.7	82.9	98.2
Kidney (n=116)	94.8	100	99.1	100
Bladder (n=35)	71.4	95.7	88.5	88.5
Esophagus (n=24)	33.3	29.2	54.1	83.3
Liver (n=42)	97.6	100	97.6	100
Pancreas (n=20)	55.0	95	95.2	100
Pleura (n=7)	42.8	85.7	100	100
Colorectal (n=57)	85.6	98.2	94.7	100
Skin (n=6)	66.6	100	100	100
Stomach (n =45)	82.2	97.8	75.5	91.1
Brain (n=47)	100	100	100	100
Cervix (n=32)	62.5	78.1	78.1	93.7
Thyroid (n=55)	98.1	100	100	100
Overall—across cancer types	84.6	95.3	96.4	97.2

**Table 2:** Performance of our deep learning model for the identification of tissue of origin on the primary tissue site cohorts from the SRA^a^.

Cancer type	SRA test accuracy—deep learning (%)
Breast (n=44)	91.6
Prostrate (n=37)	100
Lung (n=19)	100
Bladder (n=10)	80
Colorectal (n=78)	58.9
Skin (n=0)	N/A^b^
Overall—across cancer types	80.4

^a^SRA: Sequence Read Archive.

^b^N/A: not applicable.

**Table 3:** Performance of our deep learning model for the identification of tissue of origin in metastatic tumor tissue.

Cancer type	TCGA^a^ metastatic test accuracy—deep learning (%)
Breast (n=7)	85.7
Prostrate (n=1)	100
Lung (n=0)	N/A^b^
Bladder (n=1)	100
Colorectal (n=1)	100
Skin (n=352)	97.4
Overall—across cancer types	97

^a^TCGA: The Cancer Genome Atlas.

^b^N/A: not applicable.

**Table 4:** Accuracy of developed models on metastatic and SRA^a^ test sets. The accuracy for all 4 models is presented on the TCGA^b^ metastatic and SRA cohorts. The decision tree classifier had a depth of 14 and the random forest had a depth of 19.

Classifier	Accuracy on TCGA metastatic test set (%)	Accuracy on SRA test set (%)
Decision tree	62.5	41.2
Random forest	94.2	74.2
Logistic regression	93.2	71.6
Deep learning	97	80.4

^a^SRA: Sequence Read Archive.

^b^TCGA: The Cancer Genome Atlas.

These results confirm our hypotheses and show that we were able to predict the TOO with high accuracy using deep learning. Furthermore, our findings demonstrated that deep learning techniques significantly increase the accuracy in comparison to decision tree, logistic regression, and random forest models.

**Figure 5:**
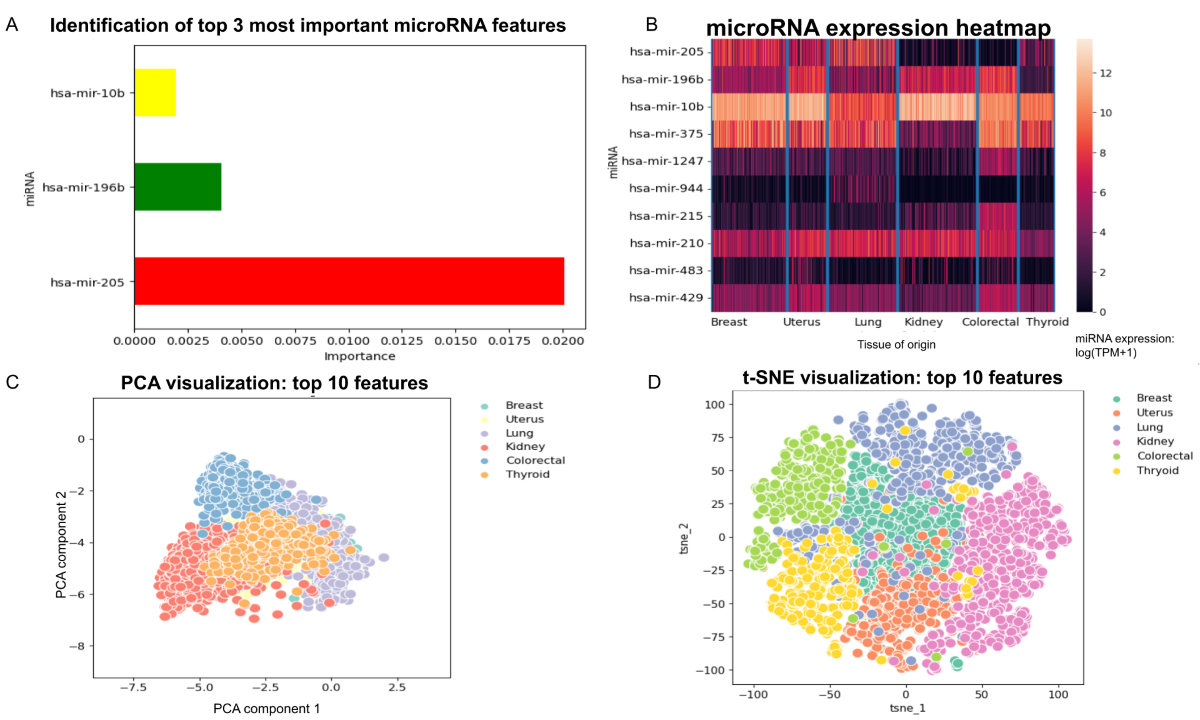
MicroRNA feature importance visualizations. (A) Permutation feature importance for the top 3 microRNA candidates. A bar graph of the importance values for the 3 top microRNA candidates for the logistic regression model. (B) MicroRNA expression heatmap. MicroRNA expression values for the top 10 most important features (as determined by permutation feature importance) for a subset of samples. The top 10 microRNA features can cluster cancer type. Low mir-205 and mir-944 and a high mir-10b are indicative of colorectal cancer. Similarly, low expressions for microRNA-429, microRNA-483, microRNA-215, microRNA-944, microRNA-1247, microRNA-375, and microRNA-205 are indicative of kidney cancer. (C) PCA visualization. (D) t-SNE visualization. PCA and t-SNE visualization of data corresponding to the 6 cancer types with the most samples in our data set, using only the top 10 microRNA features. In the PCA plot, note that there is significant overlap between the cancer types, while in the t-SNE plot, the cancer types are well separated, suggesting that with 10 microRNA features, machine learning models may correctly identify patterns and predict tissue of origin. PCA: principal component analysis; t-SNE: t-distributed stochastic neighbor embedding.

To reveal the significance of individual features, we performed feature importance analysis using the permutation feature importance method (Figure 5A). The top 3 microRNAs contributing to our deep learning model based on our combined normal and primary site training set are microRNA-10b, microRNA-196, and microRNA-205. MicroRNA-10b has been shown to function as a metastasis-promoting factor in many cancer types. In fact, it was one of the first microRNAs to have been discovered with aberrant expression in cancer cells [31]. MicroRNA-196 has been linked to the progression of many cancers, notably metastatic colorectal cancer [32], while microRNA-205 expression is downregulated in metastatic breast and prostate cancer [33].

To further understand the significance of the identified important features, we compute a heat map (Figure 5B) showing the microRNA expression values for the top 10 microRNA features for samples in the training data set. Visually, it is apparent that the microRNA features can be used to distinguish the cancer type. To further validate this, we perform principal component analysis and t-SNE analysis using only the top 10 features (Figures 5C and 5D). We note that the t-SNE plot shows a clear separation of features into distinct clusters corresponding to each cancer type, showing the significance of the features for detecting the TOO.

## Discussion

### Principal Findings

In these investigations, while using successively more powerful classifiers, we were able to detect the TOO on solely metastatic cancer samples with accuracies ranging from 62.5% (226/362) with a decision tree to 97% (351/362) with a deep learning model. Our methods show that one can leverage larger amounts of gene expression data for primary and solid tissue normal tumor samples (~10,000 samples) to come up with accurate classifiers to determine TOO for metastatic cancer (currently limited to ~300 samples). In order to verify the robustness of our model, we assessed its performance on primary tumor data from the SRA and obtained accuracies ranging from 41.2% (77/188) with decision tree to 80.4% (151/188) when using deep learning. Our methods have also identified promising microRNA candidates, reaffirming prior research in this field and demonstrating the potential of machine learning.

The predominant failure of our model on the SRA test cohort was within colorectal cancer as can be seen in Figure 4C. Many colorectal samples were incorrectly classified as stomach or gastric cancer. This is consistent with previous research in this area as microRNA expression profiles for gastrointestinal cancers show significant overlap [39]. In addition, colorectal and stomach cancer are often synchronous with probabilities ranging from 20.1% to 37.2% [40].

We used permutation feature importance, a model-agnostic metric that permutes features across samples in the test set to assess the change in model accuracy. The results are in line with existing research in this area and serve as a good indicator of the feasibility of machine learning techniques to identify promising biomarkers.

### Limitations

To effectively use our model in clinical care, accuracy must be improved further. Our model currently performs with an accuracy of 97% (351/362). While this may seem impressive, clinical classifiers should be highly accurate so that there are a negligible number of cases with errors in identifying TOO. To improve the accuracy, the accumulation of larger data sets is necessary, and as the noncoding genome continues to reveal significant contributions to cancer, we predict that available data sets will expand. A further limitation to our study is that the available microRNA metastatic data sets are predominantly skin cancer. Thus, access to a larger, more varied, data set would improve our assessment of model performance. Furthermore, in order to develop a truly noninvasive method of TOO identification relevant to all cancers, it would be ideal to extend our method to microRNA expression data from blood samples. Detecting the TOO through blood-based microRNA biomarkers would significantly impact the diagnosis and treatment of patients with CUP. Additionally, our model cannot differentiate between tumor and solid tissue normal samples, as it was designed to identify the TOO specifically.

### Conclusions

To summarize, our developed machine learning models can accurately identify the TOO with high accuracy from microRNA expression data when trained on primary tumor and solid tissue samples. Importantly, our results identified key microRNA differentiators of tissue type. Our models are robust and perform well across different data sets (TCGA and the SRA data set). We look forward to developing further deep learning models that can accurately detect TOO as microRNA data sets expand, with the goal of having a noninvasive test for diagnosing the presence of cancer and determining cancer TOO with high accuracy.

## Appendix

Multimedia Appendix 1 Detailed analyses of feature importance and confusion matrices to support the study's findings on tissue of origin classification using microRNA features.

## Abbreviations

CUP: carcinoma of unknown primary
RPM: reads per million
SRA: Sequence Read Archive
TCGA: The Cancer Genome Atlas
TOO: tissue of origin

## Data Availability

The data sets generated during and/or analyzed during this study are available in the github repository [41].
