# Supplementary material for: Deep Learning–Based Identification of Tissue of Origin for Carcinomas of Unknown Primary Using MicroRNA Expression: Algorithm Development and Validation
Source: JMIR Bioinform Biotechnol. 2024 Jul 24;5:e56538. doi: 10.2196/56538 (PMC11306940; doi:10.2196/56538)
Supplement: Multimedia Appendix 1 [file bioinform_v5i1e56538_app1.docx]

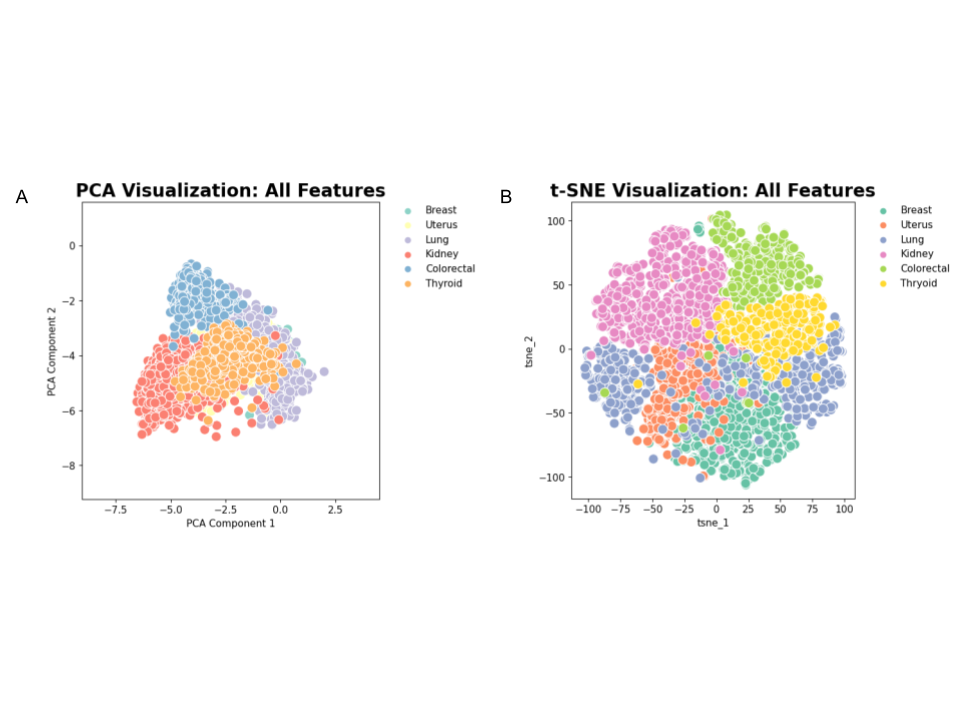


**Figure S1: PCA and t-SNE visualization for all miRNA features.** *A and B show the PCA and t-SNE visualization of data corresponding to six cancer types, using* ***all*** *miRNA features. These plots are comparable to those shown in Figure 5B and Figure 5C, showing that the top 10 miRNA features contain most of the relevant information. This shows that the permutation feature importance has successfully identified a subset of the most relevant miRNA features.*

| **Tissue of origin** | **Primary Tumor** | **Solid Tissue** | **Metastatic** | **SRA** |
| --- | --- | --- | --- | --- |
| Breast | 1096 | 104 | 7 | 48 |
| Uterus | 602 | 33 | 0 | 0 |
| Ovary | 490 | 0 | 0 | 0 |
| Prostrate | 498 | 52 | 1 | 37 |
| Testis | 150 | 0 | 0 | 0 |
| Lung | 997 | 91 | 0 | 19 |
| Kidney | 1093 | 142 | 1 | 0 |
| Bladder | 417 | 19 | 1 | 10 |
| Esophagus | 186 | 13 | 1 | 0 |
| Liver | 372 | 50 | 0 | 2 |
| Pancreas | 178 | 4 | 1 | 0 |
| Pleura | 87 | 0 | 0 | 0 |
| Colorectal | 616 | 11 | 1 | 78 |
| Skin | 97 | 2 | 352 | 0 |
| Stomach | 446 | 45 | 0 | 0 |
| Brain | 512 | 5 | 0 | 0 |
| Cervix | 307 | 3 | 0 | 0 |
| Thyroid | 506 | 59 | 0 | 0 |

**Table S1: Distribution of cancer types in TCGA (primary, solid and metastatic) and SRA datasets**

| Cancer Type | Decision Tree | Random Forest | Logistic Regression | Deep Learning |
| --- | --- | --- | --- | --- |
| Breast (n=131) | 91.6% | 99.2% | 96.9% | 99.2% |
| Uterus (n=73) | 76.7% | 100% | 90.4% | 94.5% |
| Ovary (n=48) | 89.6% | 91.6% | 93.8% | 100% |
| Prostrate (n=54) | 94.5% | 100% | 100% | 100% |
| Testis (n = 18) | 61.1% | 94.5% | 94.4% | 88.9% |
| Lung (n=117) | 81.1% | 95.7% | 82.9% | 98.2% |
| Kidney (n=116) | 94.8% | 100% | 99.1% | 100% |
| Bladder (n=35) | 71.4% | 95.7% | 88.5% | 88.5% |
| Esophagus (n=24) | 33.3% | 29.2% | 54.1% | 83.3% |
| Liver (n=42) | 97.6% | 100% | 97.6% | 100% |
| Pancreas (n=20) | 55.0% | 95% | 95.2% | 100% |
| Pleura (n=7) | 42.8% | 85.7% | 100% | 100% |
| Colorectal (n=57) | 85.6% | 98.2% | 94.7% | 100% |
| Skin (n=6) | 66.6% | 100% | 100% | 100% |
| Stomach (n =45) | 82.2% | 97.8% | 75.5% | 91.1% |
| Brain (n=47) | 100% | 100% | 100% | 100% |
| Cervix (n=32) | 62.5% | 78.1% | 78.1% | 93.7% |
| Thyroid (n=55) | 98.1% | 100% | 100% | 100% |
| **Overall - Across Cancer Types** | **84.6%** | **95.3%** | **96.4%** | **97.2%** |

**Table S2: Validation Set Accuracy for TOO classification for four ML models**


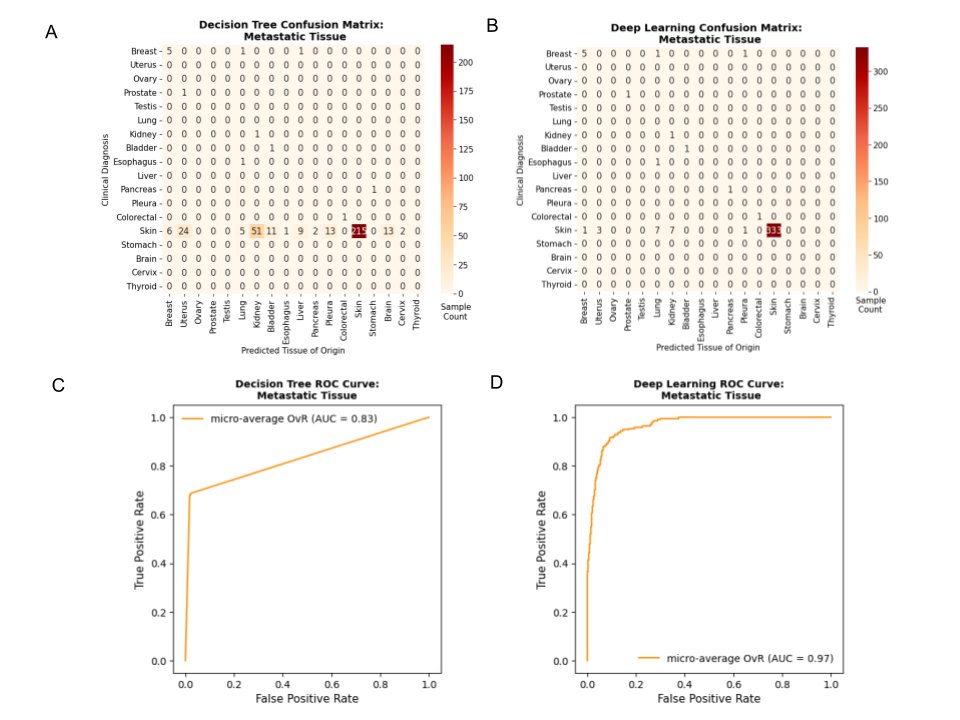


**Figure S2: Representative Confusion Matrices and ROC Curves for Created Models on TCGA Metastatic Samples.** *A) Confusion Matrices for Decision Tree and B) Deep Learning model on metastatic samples. C) ROC curves for Decision Tree and D) Deep Learning model on metastatic samples.*


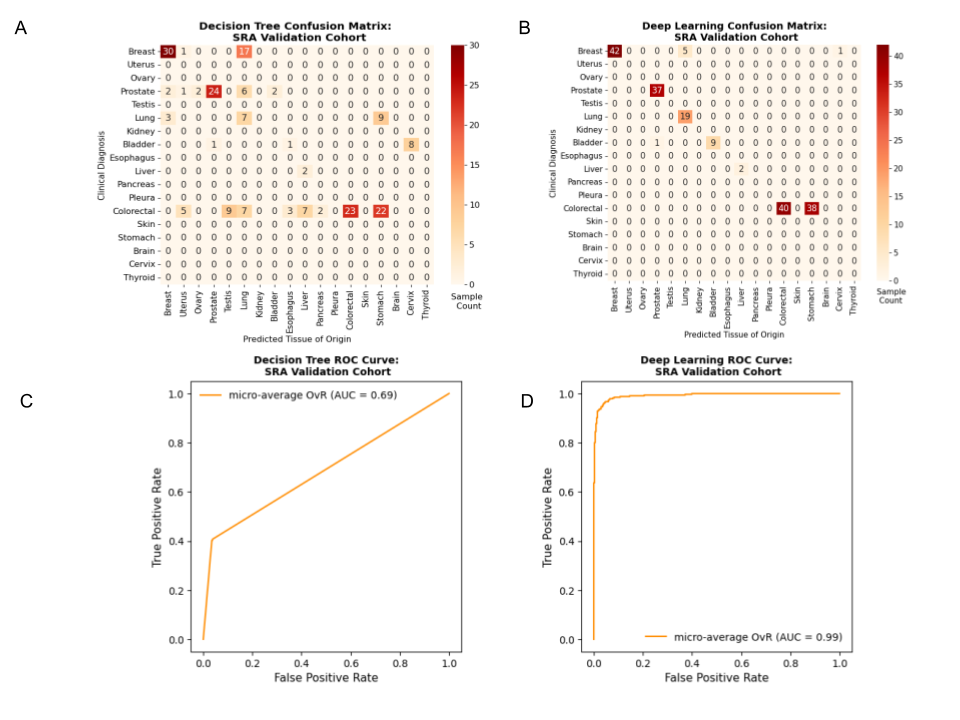


**Figure S3: Representative Confusion Matrices and ROC Curves for Created Models on SRA Samples.** *A) Confusion Matrices for Decision Tree and B) Deep Learning model on samples from SRA. C) ROC curves for Decision Tree and D) Deep Learning model on samples from SRA.*
